# Comparison of the effect of Mospilan SP and its active substance on *Artemia franciscana*

**DOI:** 10.17221/73/2025-VETMED

**Published:** 2026-05-27

**Authors:** Marcel Falis, Katarina Benova, Michaela Spalkova, Lenka Leskova

**Affiliations:** ^1^Department of Pharmacology and Toxicology, University of Veterinary Medicine and Pharmacy in Košice, Košice, Slovak Republic; ^2^Department of Biology and Physiology, University of Veterinary Medicine and Pharmacy in Košice, Košice, Slovak Republic; ^3^Department of Public Veterinary Medicine and Animal Welfare, University of Veterinary Medicine and Pharmacy in Košice, Košice, Slovak Republic

**Keywords:** LC_50_, morphological changes, neonicotinoids

## Abstract

Contaminants formed after the use of pesticides raise concerns for human health and aquatic ecosystems, i. a., due to their high persistence in surface waters and their further migration to other components of the environment. In our study, we compared the effect of the insecticide Mospilan SP and its active substance acetamiprid on the aquatic crustacean *Artemia franciscana*. The concentration was 367.44 mg/l for Mospilan SP LC_50_ after 120 h, and 102.82 mg/l after 144 hours. The concentration was 148.04 mg/l for the active substance acetamiprid LC_20_ after 144 h and 28.84 mg/l after 168 hours. The commercial insecticide Mospilan SP showed a significant suppressive effect on the measured morphological parameters (body length and body width), with statistically significant differences observed after 10 days of exposure. When comparing the changes after the application of the insecticide and the active substance, it is impossible to confirm the same effect on the morphological parameters within the given time intervals. The objective of the study was to examine morphological changes at sublethal concentrations, as these changes can provide a more sensitive indication of the adverse effects of commercial products and active substances on morphological parameters.

Pesticides have been widely used in agriculture; as their popularity increases, so does the risk of their negative effects. Given their high potential toxicity to unintended terrestrial and aquatic organisms, neonicotinoid insecticides (a class of pesticides) are classified as contaminants of concern (Rico et al. 2018). Neonicotinoid was the term introduced for imidacloprid and other insecticidal compounds whose structure and mechanism of action are comparable to the insecticidal alkaloid (*S*)-nicotine (Tomizawa and Yamamoto 1993; Tomizawa and Casida 2003; Jeschke and Nauen 2005). Upon environmental degradation, these compounds are absorbed by plants, thereby inducing insect resistance (Wexler et al. 2005).

Worldwide, neonicotinoids are the fastest-growing class of insecticides, currently approved for use on numerous field crops in over 120 nations (Morrissey et al. 2015). In 2018, the European Union banned the external use of three main neonicotinoids (clothianidin, imidacloprid, and thiamethoxam). In 2019, the United States Environmental Protection Agency (US EPA) cancelled the use of twelve products containing neonicotinoids. Nevertheless, in 35% of countries worldwide, there is no legislation on pesticides, so the mentioned neonicotinoids remain the most widely used (Klingelhofer et al. 2022).

Comparing the effects of the active substance acetamiprid with those of the product Mospilan SP at sublethal concentrations – specifically, below the maximum tolerated dose threshold – is an optimal approach for identifying harmful effects of these substances. This method allows the detection of adverse impacts that may be detrimental, even if they do not result in lethality.

Aquatic organisms are often exposed to contaminants in their natural habitats. However, the toxic effects of chemical substances on these organisms and how they cope with them remain poorly understood (Hu et al. 2022). Since 2010, neonicotinoids, one of the most important classes of insecticides, have become widely criticised for their possible negative or even toxic effects on pollinators, especially honeybees and wild bees (Goulson et al. 2018). The occurrence of emerging contaminants, such as pesticides, and their degradation products in the environment also raises concerns for human health and aquatic ecosystems, for instance, due to their high persistence in surface waters and their further migration into other environmental components. Therefore, we have chosen the aquatic crustacean *A. franciscana* as a model organism. The toxicity, potential bioaccumulation in the environment, or leaching of insecticides from the soil into the groundwater are just a few examples of why neonicotinoids need to be studied more closely (Pietrzak et al. 2020). One of the neonicotinoids is Mospilan SP containing the active substance acetamiprid. It has been widely used against various pests, such as aphids, mealybugs, potato armyworms, and many others, not only by farmers but also by gardeners and small growers.

## MATERIAL AND METHODS

The concentrations used in the test correspond to the concentrations of the product and the active substance in the spray liquid. Based on the approved uses for individual crops and recommended application methods, the concentration of the Mospilan 20 SP product in the spray liquid ranges from 250 mg/l to 1 250 mg/l, corresponding to an active substance concentration of 50 mg/l to 250 mg/l. These concentrations are used in the study.

To determine the LC_50_ during the trial, a 10-day test with *A. franciscana* was used (Dvorak et al. 2005). Investigations were carried out with Mospilan^®^ (active compound acetamiprid) at a concentration of 7.5 mg/l, 15.0 mg/l, 30.0 mg/l, 60.0 mg/l, 120.0 mg/l, and 240.0 mg/l. Nauplia were randomly divided into seven groups – one control, and six experimental groups. Each group comprised 50 nauplia subdivided into five separate subgroups (dishes), ten individuals in each. In total 350 nauplia were investigated.

Investigations were carried out with acetamiprid at a concentration of 6.25 mg/l, 12.5 mg/l, 25.0 mg/l, 50.0 mg/l, 100.0 mg/l, and 200.0 mg/l. Nauplia were randomly divided into eight groups– two control [one group with the addition of dimethyl sulfoxide (DMSO)], the second one without the addition), and six experimental groups. Each group comprised 50 nauplia subdivided into five separate subgroups (dishes), ten individuals in each. In total 400 nauplia were investigated.

The Petri dishes were placed into a previously disinfected thermostat set to 20 (±1) °C. Checks for immobilisation and lethality were performed every 24 h after the test began, with the results then compared to those found in the untreated control groups (OECD 2004). The statistical determination of the EC_10_, EC_20_, EC_50_, LOEC and NOEC values (EC = effect concentrations) at 48 and 96 h was carried out by the statistical software ToxRatPro^®^ v3.3.0 (ToxRat Solutions GmbH, Alsdorf, Germany).

For the morphology assessment, a 10-day test for *A. franciscana* was used (Dvorak et al. 2005). The Mospilan concentrations were 7.5 mg/l, 15.0 mg/l, 30.0 mg/l, 60.0 mg/l, and 120.0 mg/l. The acetamiprid concentrations were 3.125 mg/l, 6.25 mg/l, 12.5 mg/l, 25.0 mg/l, and 50.0 mg/l. With the acetamiprid trial, only the control group with DMSO was used. 150 individuals were used in each group (1 800 in total). Every 24 h, we transferred individuals from the plates intended for morphology assessment into wells on a slide using a pipette. To euthanise the observed individuals, DMSO was dripped into the wells on the slide. For the measurements, we used an Olympus CS 23 trinocular microscope equipped with a camera projecting the image in real-time to the computer. The following parameters were assessed: body length and body width. In the statistical analysis, both the arithmetic mean and standard deviation for all monitored parameters are consistently included in the figures and tables. To determine the relationship between the monitored parameters (e.g., body dimensions) and various doses, a one-factor analysis of variance (ANOVA) was conducted. This analysis utilised significance levels of α = 0.05, α = 0.01, and α = 0.001. The statistical data were generated using ToxRat Professional software, v3.3.0, released on 20 October 2018.

## RESULTS

### LC_50_ Mospilan

**Figure 1:**
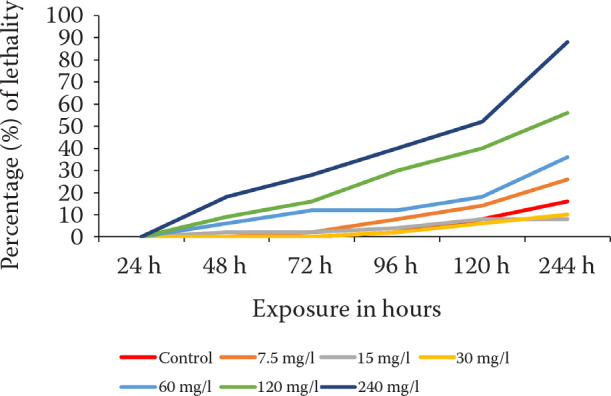
Lethality of *Artemia franciscana* after exposure to Mospilan

Figure 1 shows the lethality of *A. franciscana* after the effect of Mospilan SP concerning the time and concentration. For Mospilan, the LC_50_ after 120 h was 367.44 mg/l, and it was 102.82 mg/l after 144 h (ToxRat software) (Table 1).

**Table 1:** Summary of the results for Mospilan: Critical effect and threshold concentration as observed at the end of experimental time

Critical concentrations (mg/l)		Immobility					
		0–24 h	0–48 h	0–72 h	0–96 h	0–120 h	0–144 h
95%-CL	LC_10_	nd	158.09	83.47	39.01	18.53	11.12
	lower	nd	87.16	49.63	0.16	nd	nd
	upper	nd	427.44	124.98	97.62	51.80	nd
							
95%-CL	LC_20_	nd	458.74	191.47	100.97	51.66	23.86
	lower	nd	222.67	127.72	23.61	1.150	nd
	upper	nd	4 174.25	360.36	941.32	177.73	nd
							
95%-CL	LC_50_	nd	nd	nd	nd	367.44	102.82
	lower	nd	nd	nd	nd	125.44	nd
	upper	nd	nd	nd	nd	nd	nd
							
Immobility	LOEC	nd	120.00	60.00	120.00	120.00	120.00
	NOEC	nd	60.00	30.00	60.00	60.00	60.00

95%-CL = 95% confidence limit; LC = effective concentration for xx% reduction; LOEC = lowest observed effect concentration; nd = not determined due to mathematical reasons or inappropriate data; NOEC = no observed effect concentration

### LC_50_ Acetamiprid

**Figure 2:**
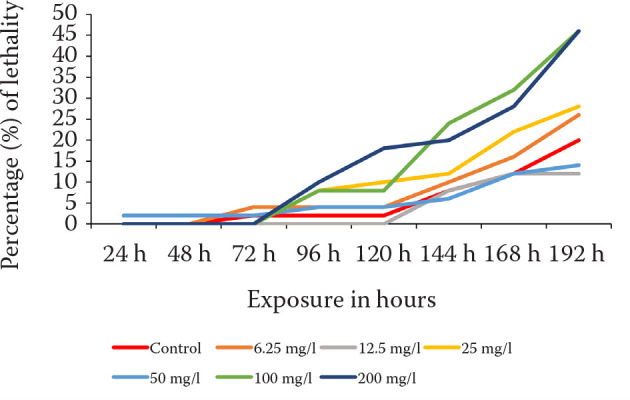
Lethality of *Artemia franciscana* after exposure to the active substance acetamiprid

As presented in Figure 2, for the active substance acetamiprid, we did not record the 50% lethality because the maximum tested dose was 200 mg/l (solubility issues). Using the ToxRat software, the LC_50_ was calculated as 148.04 mg/l after 144 h and 28.84 mg/l after 168 h (Table 2).

**Table 2:** Summary of the results for acetamiprid: Critical effect and threshold concentration as observed at the end of experimental time

Critical concentrations (mg/l)		Immobility						
		0–24 h	0–48 h	0–72 h	0–96 h	0–120 h	0–144 h	0–168 h
95%-CL	LC_10_	nd	nd	nd	174.54	69.07	12.38	nd
	lower	nd	nd	nd	nd	29.68	nd	nd
	upper	nd	nd	nd	nd	415.81	34.32	nd
								
95%-CL	LC_20_	nd	nd	nd	nd	nd	148.04	28.84
	lower	nd	nd	nd	nd	nd	50.77	2.74
	upper	nd	nd	nd	nd	nd	nd	152.04
								
95%-CL	LC_50_	nd	nd	nd	nd	nd	nd	nd
	lower	nd	nd	nd	nd	nd	nd	nd
	upper	nd	nd	nd	nd	nd	nd	nd
								
Immobility	LOEC	nd	nd	200.00	200.00	200.00	200.00	nd
	NOEC	nd	nd	200.00	200.00	100.00	200.00	nd

95%-CL = 95% confidence limit; LC = effective concentration for xx% reduction; LOEC = lowest observed effect concentration; nd = not determined due to mathematical reasons or inappropriate data; NOEC = no observed effect concentration

### Mospilan

When evaluating body length, we did not observe any obvious trends in variation of the parameter across the concentration range of 7.5–60 mg/l from Day 1 to Day 6 of exposure. After Day 8 of exposure, at a dose of 120 mg/l across all time frames, we observed a statistically significant decrease in body length in most cases.

**Figure 3:**
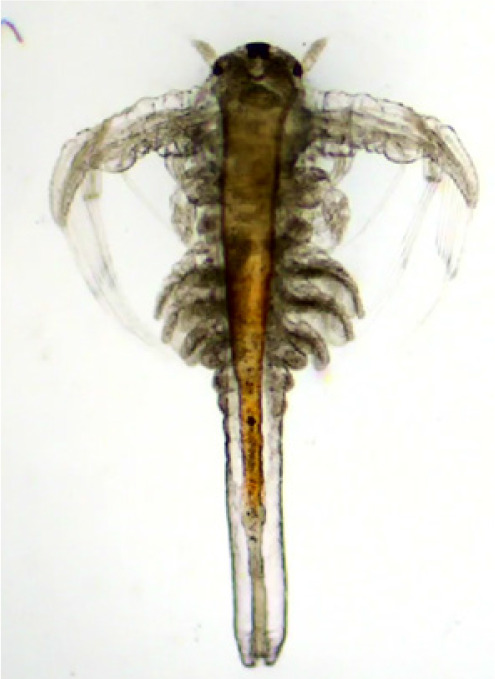
An individual from the control group at Day 8

**Figure 4:**
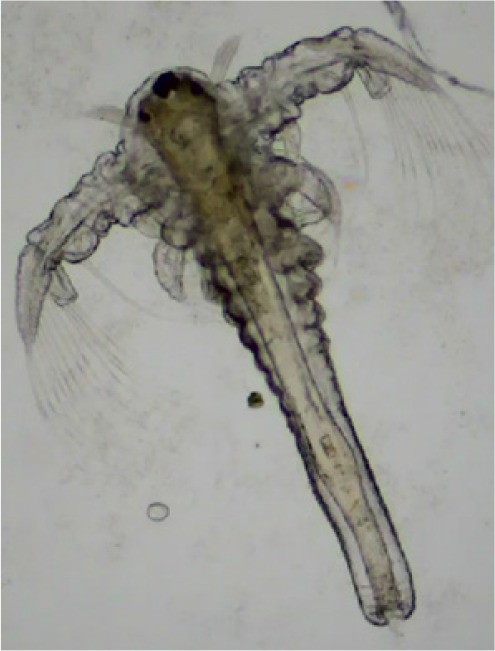
An individual from the group at a dose of 120 mg/l at Day 8 – a marked decrease in pleopod development compared to the control

When evaluating the body width, in the concentration range of 7.5–60 mg/l in the time frame from Day 1 to Day 8 of exposure, we did not record any continuing trends in the variation of the given parameter, although on Day 6 of exposure, we observed a statistically significant decrease in all the concentrations. On Day 8 of exposure, at the dose of 120 mg/l (in all the time intervals), we noticed a decrease in the body width in all the cases at a statistically significant level (Figure 3 and Figure 4).

### Acetamiprid

When evaluating body length, we did not observe a noticeable trend in variation of the given parameter across the concentration range of 3.125–50 mg/l over the exposure period from Day 1 to Day 8. On Day 10 of exposure, we observed a statistically insignificant decrease in body length across all concentrations.

When evaluating the body width, we observed a decrease in this parameter at all the concentrations on Day 6. On Day 10, we observed a statistically insignificant increase in the given parameter in the concentration range of 3.125–25 mg/l. In the other cases, no visible effect of acetamiprid on the given parameter was observed.

## DISCUSSION

The EU’s efforts to reduce the number of experimental vertebrates can be addressed by replacing conventional tests with alternative biotests. Biotests on planktonic organisms (*Artemia franciscana*), unlike isolated tissue cultures, represent a model based on a complex living organism. These experiments more closely resemble natural conditions than those conducted on special lines of experimental vertebrates (Dvorak et al. 2010).

Pesticides are defined as xenobiotics used mainly in agriculture to prevent, destroy, or repel pests. Based on the species they target, pesticides are broadly categorised into insecticides, herbicides, fungicides, and rodenticides, with additional, less prevalent groups including acaricides, molluscicides, larvicides, pediculicides, viricides, and bactericides. Neonicotinoids are a new generation of widely used insecticides. They act on the post-synaptic nicotinic acetyl-cholinergic receptors (nAChR) of an insect as antagonists of the receptors that are exclusively found in the central nervous system (CNS), leading to repeated spasms and subsequent paralysis due to the complete blockage of nerve impulses (Simon-Delso et al. 2015). Neonicotinoids are characterised by a higher affinity to insect receptors versus mammalian ones, have high efficiency, and require simple manipulation. Acetamiprid, the active substance of Mospilan^©^ 20 SP used in our study, is a representative of this group of insecticides.

According to the manufacturer’s Material Safety Data Sheet (MSDS) for Acetamiprid 20 SP, the reported toxicity for *Daphnia* spp. is a 24-hour LC_50_ greater than 200 mg/l and a 48-hour EC_50_ of 49.8 mg/l, while for bees, the oral LD_50_ is 14.5 µg/bee and the contact LD_50_ is 8.1 µg/bee (UPI Cropscience 2022).

Acetamiprid itself did not dissolve in the salt water used in the experiment, so 1% DMSO was used to facilitate dissolution. Although the works of other authors indicate that low concentrations of DMSO do not affect lethality or morphology (Chen et al. 2011; Luptakova et al. 2021), we used control groups with and without DMSO; we did not observe a statistically significant difference in either.

The effects of neonicotinoids have recently been monitored in many species, including bees, frogs, silkworms, fish, and others. The effect varied in different organisms; it was also dependent on the used concentration during the experimental exposure.

Neonicotinoids were studied for their effects on the juvenile stages of two mollusc species: *Lampsilis fasciola* (*Unionidae*) and *Planorbella pilsbryi* (*Planorbidae*). *P. pilsbryi* juveniles were exposed for 7 or 28 days to thiamethoxam, imidacloprid, or clothianidin. The biomass production, growth, and mortality were assessed. *L. fasciola* larvae were exposed for 48 h to various neonicotinoids (thiacloprid, thiamethoxam, acetamiprid, clothianidin, dinotefuran, or imidacloprid), and their viability was analysed. The studies found that the growth and biomass production were more sensitive indicators of the neonicotinoid impact than the mortality. Overall, the neonicotinoid exposure appeared to pose a lower risk to these molluscs compared to the potential vulnerability observed in non-target aquatic insects (Prosser et al. 2016).

Crustaceans showed a level of sensitivity similar to that of insects, with an EC_50_ of less than one part per million (ppm). In contrast, midge larvae were less sensitive than other insects, despite also having an EC_50_ below 1 ppm. The most sensitive reaction was observed in the insect *Chironomus riparius* (*Chironomidae*), which exhibited a NOEC of 0.01 ppm after 30 days of exposure (Malhotra et al. 2021). Similar levels of toxicity were observed in both marine and freshwater organisms (Finnegan et al. 2017).

In a study by Rico et al. (2018), aquatic invertebrates were exposed to either an equimolar mixture of five neonicotinoids (acetamiprid, imidacloprid, thiacloprid, thiamethoxam, and clothianidin) or a single dose of imidacloprid. The highest sensitivity to neonicotinoids was observed at a NOEC of less than 0.2 ppb (parts per billion) for *Cyclopoida*, *Cloeon deipterum* (*Baetidae*), and *Chironomini* spp. (*Chironomidae*). An interesting finding was that the short-term effects of the neonicotinoid mixture and the single imidacloprid concentration on the macroinvertebrate and zooplankton communities were comparable. This suggests that the concentration addition model could be a reasonable hypothesis for predicting the effects of neonicotinoid mixtures in aquatic ecosystems. However, the study emphasised that long-term mixture toxicity assessments are necessary to fully understand the environmental impact of these substances.

The effects of acetamiprid on honeybees (*Apis mellifera*) include metabolic disorders of bee larvae at concentrations higher than 5 mg/l (Shi et al. 2023), the shortening of the life cycle, reducing the flight activity of worker bees, changing the foraging behaviour (Shi et al. 2020), and locomotion disorders in older bees (Aliouane et al. 2009).

Morphological changes after the effect of neonicotinoids were also described by some authors. A similar inhibitory effect on growth (body length), as in our study, was observed in the study by Ma et al. (2019) in Zebrafish embryos, with increasing concentrations of another neonicotinoid (imidacloprid) after 120 h of exposure (Table 3).

**Table 3:** Summary of the measured body size parameters after exposure to Mospilan SP

Parameter evaluated	Concentration (mg/l)	Day 1	Day 3	Day 6	Day 8	Day 10
Body length (µm ± SD)	0 (control)	343.55 ± 12.27	445.53 ± 10.67	476.50 ± 24.91	541.06 ± 7.28	602.49 ± 15.00
	7.5	323.64** ± 5.96	418.63 ± 16.75	483.22 ± 15.12	494.21** ± 13.19	493.55* ± 32.66
	15	331.24 ± 23.3	436.43 ± 22.74	490.29 ± 37.61	509.54* ± 19.14	524.76* ± 15.83
	30	345.80 ± 21.89	464.26 ± 28.20	481.22 ± 15.64	490.11* ± 40.07	499.79* ± 28.5
	60	327.09 ± 23.01	444.11 ± 23.53	521.71 ± 31.09	538.11 ± 21.08	579.01 ± 50.82
	120	344.20 ± 30.89	369.16* ± 18.17	383.54*** ± 23.13	383.28* ± 38.93	–
						
Body width (µm ± SD)	0 (control)	44.53 ± 1.01	99.87 ± 6.71	116.81 ± 1.90	105.06 ± 2.70	124.67 ± 7.12
	7.5	43.24 ± 5.98	92.64 ± 3.30	109.49* ± 5.95	101.85 ± 4.44	106.27* ± 6.19
	15	47.84 ± 4.55	88.95 ± 8.73	100.20* ± 5.19	115.30 ± 4.45	114.41* ± 4.11
	30	40.35** ± 2.39	100.54 ± 8.69	99.61* ± 3.48	99.44 ± 6.61	107.92* ± 8.63
	60	45.49 ± 6.64	89.12* ± 5.93	108.60* ± 4.98	117.84 ± 2.92	116.14* ± 5.97
	120	39.95* ± 4.17	67.48* ± 3.26	72.12* ± 6.32	76.35** ± 10.37	–

Statistically significant differences are marked in bold

*P < 0.05; **P < 0.01; ***P < 0.001

Similar results were reported by Bartlett et al. (2019), who examined the effect of acetamiprid on the growth of *Hyalella azteca*; they observed growth reduction at concentrations lower than those that affected individual survival (Table 3).

The selected concentrations were based on the composition of the spray solution, even though these levels would not normally be encountered in the environment. The Scientific Committee on Health, Environmental and Emerging Risks has recommended a maximum environmental concentration of 0.16 µg/l (SCHEER 2022). To properly assess sublethal effects, standard testing guidelines require that concentrations are chosen to produce mortality rates ideally not exceeding 10%; the concentration range used in this study was selected to meet this criterion.

When comparing morphological changes caused by Mospilan and the active substance acetamiprid (considering the 20% content of the active substance), 120 mg/l is equal to approximately the concentration of 25 mg/l of acetamiprid (60 mg/l = 12.5 mg/l; 30 mg/l = 6.25 mg/l; 15 mg/l= 3.125 mg/l).

When comparing the changes in morphological parameters induced by the commercial preparation versus those induced by the active substance alone, it is not possible to confirm identical effects within the given time intervals (Table 3, Table 4). This indicates that the effect is more pronounced when testing the commercial product, suggesting that, in certain cases, the influence of auxiliary substances – co-formulants – may be more significant than that of the active ingredient itself. It is necessary to consider these differences when assessing the risks of specific preparations, as the manifestations of toxicity and adverse effects may also differ. In our study, we also observed a more pronounced effect on changes in morphological parameters with Mospilan SP compared to its active ingredient, acetamiprid.

**Table 4:** Summary of the measured body size parameters after exposure to the active substance of acetamiprid

Parameter evaluated	Concentration (mg/l)	Day 1	Day 3	Day 6	Day 8	Day 10
Body length (µm ± SD)	0 (control)	332.02 ± 2.53	418.55 ± 13.47	449.12 ± 12.87	461.62 ± 25.29	440.99 ± 23.40
	3.125	361.51 ± 20.98	408.53 ± 22.88	475.15 ± 23.99	459.38 ± 19.78	484.43 ± 28.54
	6.25	343.86 ± 22.80	437.11 ±.21.72	470.78 ± 24.41	463.26 ± 11.59	490.24 ± 28.26
	12.5	331.17 ± 21.48	416.75 ± 22.60	465.40 ± 21.15	435.78** ± 15.89	491.16 ± 17.66
	25	389.85 ± 24.30	450.31 ± 14.26	415.44*** ± 11.91	461.33 ± 7.91	499.14 ± 23.87
	50	347.61 ± 15.04	408.22 ± 22.61	451.18 ± 7.03	424.86*** ± 13.31	457.79 ± 40.23
						
Body width (µm ± SD)	0 (control)	77.42 ± 2.36	92.78 ± 2.86	102.39 ± 7.11	100.65 ± 7.20	89.03 ± 5.13
	3.125	77.38 ± 5.20	95.92 ± 4.00	101.48 ± 2.03	103.19 ± 4.55	104.26 ± 1.24
	6.25	78.28 ± 2.95	90.85 ± 6.17	100.64 ± 1.52	101.10 ± 2.88	96.08 ± 10.17
	12.5	80.08 ± 8.98	91.86 ± 6.4	100.59 ± 5.52	97.38 ± 1.00	101.09 ± 2.06
	25	78.46 ± 3.03	103.74 ± 3.17	89.63*** ± 2.51	99.01 ± 2.77	100.60 ± 4.68
	50	82.88 ± 5.83	93.91 ± 7.27	95.96 ± 8.25	94.85 ± 2.99	89.01 ± 10.85

Statistically significant differences are marked in bold

*P < 0.05; **P < 0.01; ***P < 0.001
